# Meta-analytic approach for transcriptome profiling of herpes simplex virus type 1

**DOI:** 10.1038/s41597-020-0558-8

**Published:** 2020-07-09

**Authors:** Dóra Tombácz, Gábor Torma, Gábor Gulyás, Norbert Moldován, Michael Snyder, Zsolt Boldogkői

**Affiliations:** 1 Department of Medical Biology, Faculty of Medicine, University of Szeged, Somogyi B. u. 4., 6720 Szeged, Hungary; 2 Department of Genetics, School of Medicine, Stanford University, 300 Pasteur Dr, Stanford, California, USA

**Keywords:** Herpes virus, Next-generation sequencing, RNA sequencing, Transcriptomics

## Abstract

In this meta-analysis, we re-analysed and compared herpes simplex virus type 1 transcriptomic data generated by eight studies using various short- and long-read sequencing techniques and different library preparation methods. We identified a large number of novel mRNAs, non-coding RNAs and transcript isoforms, and validated many previously published transcripts. Here, we present the most complete HSV-1 transcriptome to date. Furthermore, we also demonstrate that various sequencing techniques, including both cDNA and direct RNA sequencing approaches, are error-prone, which can be circumvented by using integrated approaches. This work draws attention to the need for using multiple sequencing approaches and meta-analyses in transcriptome profiling studies to obtain reliable results.

## Introduction

Second-generation short-read sequencing (SRS) technology -launched in the mid-2000s-, has revolutionized both genomic and transcriptomic research because of its ability to sequence millions of nucleic acid fragments simultaneously at a relatively low expenditure per base. Third generation long read sequencing (LRS) approaches have emerged in recent years. Currently, two LRS methods are in use: single-molecule real-time technology and nanopore-based sequencing developed by Pacific Biosciences (PacBio) and by Oxford Nanopore Technologies (ONT), respectively.

LRS can overcome several shortcomings of SRS in transcriptome analysis mainly based on the ability of LRS techniques to read full-length RNA molecules. However, similarly to SRS, LRS techniques often produce spurious transcripts owing to issues such as template switching and mispriming in reverse transcription (RT) and PCR. The major problem is that no efficient bioinformatic tools are currently available to detect these errors. Direct RNA (dRNA) sequencing is considered superior to cDNA sequencing because dRNA sequencing does not involve RT, second strand synthesis and amplification by PCR, which are prone to generate artefacts (however, notably, direct cDNA sequencing without PCR amplification is also possible using both LRS platforms). Nonetheless, dRNA-Seq has also limitations, such as low throughput, 15–30 bases and missing from the transcription start site. Moreover, errors produced by, for example the ligation used for the attachment of adapters, single-strand cDNA formation, or the potential slippage of RNA molecules during their passage across the nanopore as a result of temporary improper functioning of the ratcheting enzyme are also noteworthy drawbacks of dRNA-seq. The low throughput of dRNA-Seq makes both transcript identification and the annotation of nucleic acid sequences at base-pair resolution difficult, which is especially critical in species with large genomes. LRS has already been applied for the transcriptome analysis of various organisms^1,2^, including viruses^3–9^. This approach has revealed extremely complex transcriptome profiles in every examined species. LRS techniques can be used in analyses that are challenging for SRS approaches, such as the detection of multi-spliced transcripts, parallel transcriptional overlaps, low-abundance transcripts, very long RNA molecules and embedded transcripts, including 5′-truncated ORF-containing mRNAs and non-coding transcripts. A single technique may fail to detect certain transcripts or transcript isoforms, and to precisely map transcript ends or intron boundaries. Additionally, platform- and library preparation-dependent sequencing errors may produce false isoforms. A meta-analysis including multiplatform approaches, such as various LRS and SRS techniques, as well as different auxiliary methods, such as cap selection, and 5′- and 3′-ends mapping can circumvent the aforementioned problems, especially if different library preparation protocols are used. Furthermore, the comparison of various datasets provides a tool for identifying novel transcripts, validating already-described RNA molecules or removing putative transcripts if not confirmed by other techniques.

Herpes simplex virus type 1 (HSV-1) is a member of *Alphaherpesvirinae* subfamily of the *Herpesviridae* family. According to estimates by the WHO, more than 3.7 billion people are infected with this virus worldwide^10^. HSV-1 has a 152-kbp linear double-stranded DNA genome, which is transcribed by the host RNA polymerase in a cascade-like manner producing three kinetic classes of transcripts and proteins: immediate-early (IE), early (E), and late (L)^11^. IE genes code for transcription activators required for the expression of E and L genes. The viral E genes primarily specify proteins playing a role in DNA synthesis, whereas L genes encode structural proteins. The identification of HSV-1 transcripts faces an important challenge due to the polycistronic and overlapping nature of viral transcripts. However, polycistronic units of herpesviruses are different from those of bacterial operons, in that only the most upstream genes are translated due to the use of cap-dependent translation initiation^12^. The herpesvirus genes are organized into tandem clusters generating transcripts with co-terminal transcription end sites (TESs). Previous studies have revealed several novel mRNAs, long non-coding RNAs (lncRNAs)^13–17^ and microRNAs^18^.

## Results

**Fig. 1:**
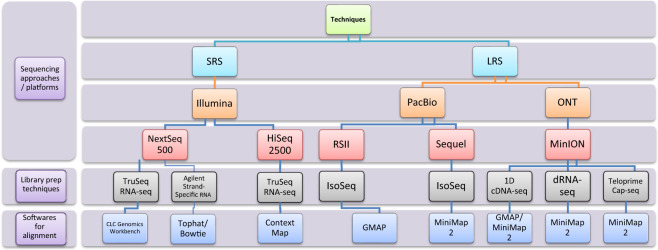
Methodological workflow shows a detailed overview of the various techniques used in the studies chosen for this meta-analysis. SRS: short-read sequencing; LRS: Long-read sequencing; PacBio: Pacific Biosciences; ONT: Oxford Nanopore Technologies.

**Fig. 2:**
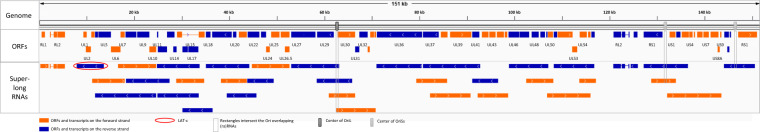
Super-long transcripts of herpes simplex virus type 1. These large (≥4 kbps) RNA molecules were identified using ONT MinION dRNA-Seq and PacBio Sequel techniques. Many of them have uncertain TSSs, especially those ones which were detected by dRNA-Seq. Only the longest transcripts are illustrated at a certain genomic region, except for overlapping transcripts which are complementary to each other.

**Fig. 3:**
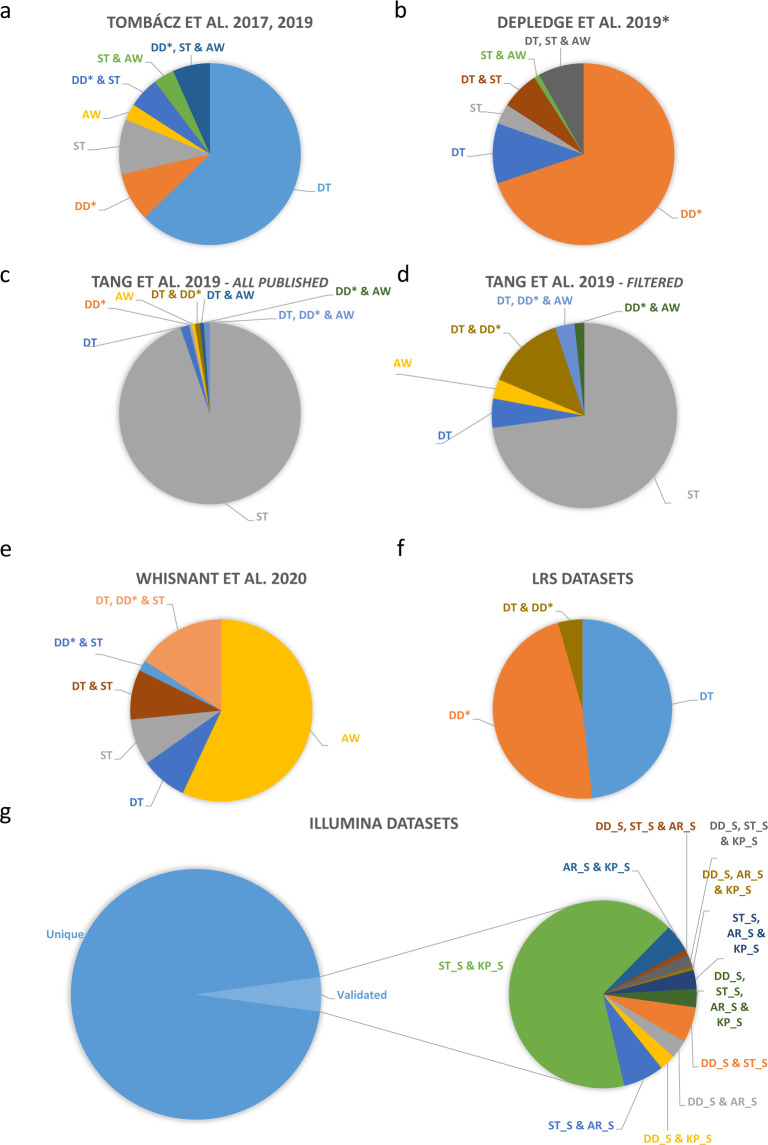
Herpes simplex virus type 1 (HSV-1) introns identified using different sequencing platforms. The 378 putative introns identified in our earlier study^25,27^ are already multiplatform-based (various combinations of library preparation techniques of Pacific Biosciences RSII and Sequel, and Oxford Nanopore Technologies MinION sequencing). These datasets were compared with the intron datasets generated by Tang *et al*.^20^ and Whisnant *et al*.^22^. We also used raw sequencing reads from Depledge’s direct RNA-Seq study^19^. The obtained data were aligned to the HSV-1 genome and then analysed using LoRTIA. This analysis detected 214 introns. Four large raw Illumina datasets^19–22,24^ were also mapped and reanalysed. Only the introns that were present in at least two independent datasets were accepted and plotted. We obtained 3,848 additional potential introns from this part of the work (see in Supplementary Table 2). **(a)** Introns identified by Tombácz and colleagues. Altogether, 44.7% of these introns have been validated by the other studies. (**b)** Introns identified in Depledge and co-workers’ dataset using the LoRTIA tool. Our analysis of the raw dRNA-Seq reads detected 309 potential introns, from which 104 were also found in the other studies. The LoRTIA tool did not identify the previously published intron within the RNA encoding the fusion protein RL2–UL1^19^; however, it was verified by the dataset from Tang and colleagues’ publication^20^. (**c)** Introns published by Tang and colleagues. These authors published a large number of potential introns (2352), but only 5% of them were validated in the other datasets. (**d)** High-coverage introns from Tang and co-workers’ publication. 59 out of 2,352 detected introns were identified as highly abundant by the authors. From these 59, only 16 (27%) were detected in at least one of the other three published intron datasets. **(e)** Introns from Whisnant and colleagues’ publication. They have published 79 introns, 84% of which were also found in other datasets. The authors have analysed our previous dataset^22^ and found that seven of the eleven published introns are low-abundance isoforms. Therefore, they considered them as unconfirmed. We found and validated five out of these seven introns in our novel dataset, which were also present in Tang’s and/or Depledge’s datasets. **(f)** Distribution of the introns identified only in LRS dataset(s). Our analysis identified more than 400 potential introns which were not validated by the analysis of either Illumina dataset. Five per cent of these introns were found in both LRS data. **(g**) Reanalysis of HSV datasets from various Illumina sequencing experiments. This work yielded 4,180 introns which were detected in at least two of the datasets. DT: Tombácz *et al*. 2017 & 2019; DD: Depledge *et al*. 2019; ST: Tang *et al*. 2019; AW: Whisnant *et al*. 2019 & 2020; AR_S: dataset from Rutkowski *et al*. 2019 analysed by STAR; DD_S: Illumina dataset from Depledge *et al*. 2019 analysed by STAR; KP_S: dataset from Pheasant *et al*. 2018; ST_S: dataset from Tang *et al*. 2019 analysed by STAR.

**Fig. 4:**
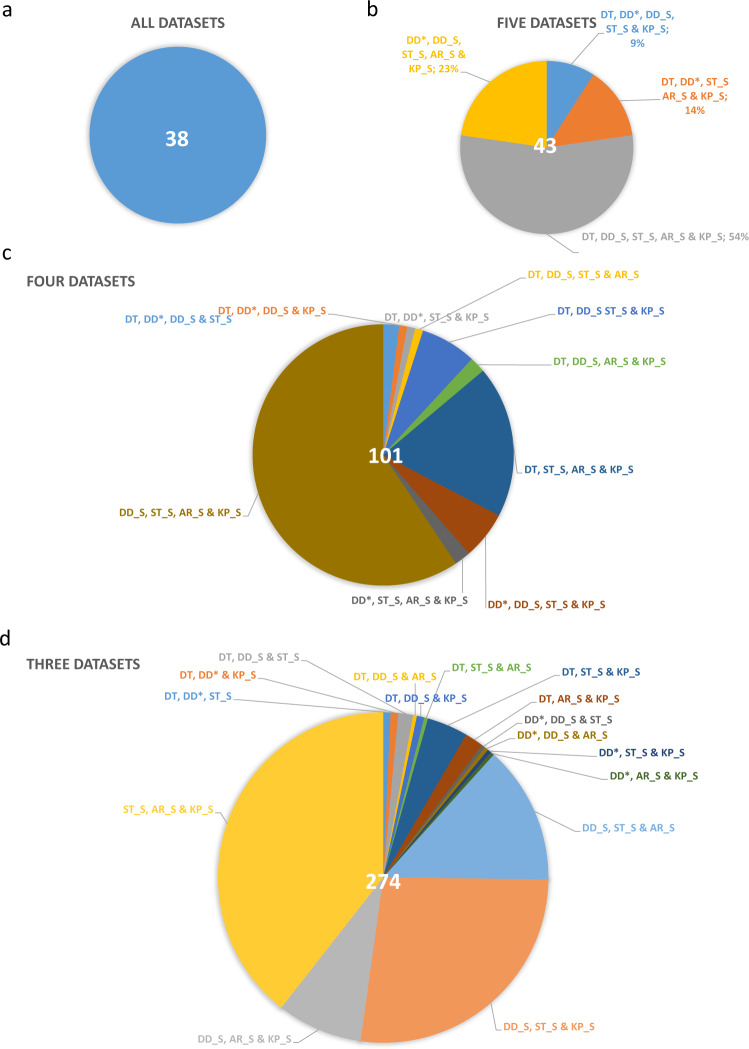
Introns, identified in at least three independent experiments. **(a)** Thirty-eight introns were detected in all six datasets. (**b**) Introns validated by five experiments. The largest “five-dataset” combination (56%) contains introns which were detected in the four Illumina datasets and the dataset from our laboratory. (**c**) Five dataset validated introns. 59% of these introns were detected within the four Illumina datasets. (**d**) 274 introns were shown in 3 independent experiments. DT: Tombácz *et al*. 2017 & 2019; DD: Depledge *et al*. 2019; ST: Tang *et al*. 2019; AW: Whisnant *et al*. 2019 & 2020; AR_S: dataset from Rutkowski *et al*. 2019 analysed by STAR; DD_S: Illumina dataset from Depledge *et al*. 2019 analysed by STAR; KP_S: dataset from Pheasant *et al*. 2018; ST_S: dataset from Tang *et al*. 2019 analysed by STAR.

**Fig. 5:**
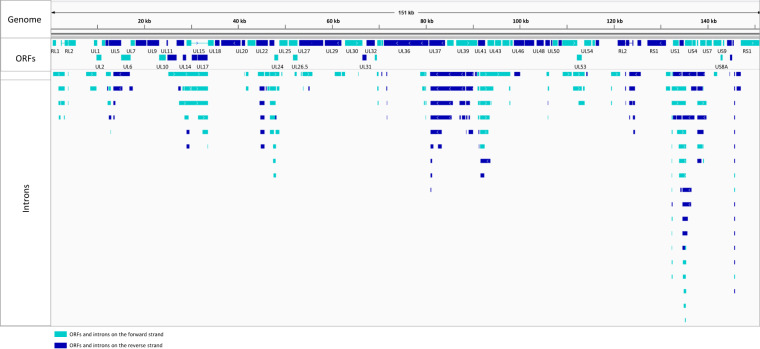
Integrative Genomics Viewer representation of the intron positions.

In this study, we employed an integrated approach based on the meta-analysis of the HSV-1 transcriptome data published by Depledge and colleagues (using ONT dRNA-Seq and Illumina RNA-Seq)^19^, Tang *et al*. (using Illumina SRS)^20^, Rutkowski *et al*. (using Illumina SRS)^21^, Wishnant *et al*. (using Illumina SRS)^22,23^, Pheasant *et al*. (using Illumina SRS)^24^ and our laboratory (Tombácz and colleagues using PacBio RSII^25^, as well as Boldogkői *et al*.^26^, and Tombácz *et al*.^27^ using PacBio Sequel, ONT dRNA-Seq and cDNA sequencing with multiple library preparation methods; Fig. 1, Supplementary Table 1). Our investigations led to the discovery of several novel transcripts, especially of novel multigenic RNA molecules (Fig. 2), and novel splice sites (Figs. 3–5; Tables 1 and 2, and Supplementary Tables 2 and 3). As Figs. 3 and 4 show, a relatively high percentage of introns identified in a study were not detected in other studies, probably due to the varying strictness of criteria used for the annotations. Another possible reason for the large number of unique introns may derive from the variance between the methodologies (e. g. viral titre of infection, virus strain, etc.) used for dataset generation. We note that a large number of unique introns share the splice donor and/or acceptor sites with other introns, which suggests the existence of these splice sites.

**Table 1:** Introns identified in all datasets.

Intron positions		DNA strand	Intron motif	Intron length
2318	3082	+	GT/AG	764
3750	3885	+	GT/AG	135
3750	3888	+	GT/AG	138
12179	12299	−	CT/AC	120
12429	12971	+	GT/AG	542
13449	13931	−	CT/AC	482
29068	29661	−	CT/AC	593
30049	33634	+	GT/AG	3585
41710	42245	+	GT/AG	535
46772	48031	+	GT/AG	1259
46772	48074	+	GT/AG	1302
46772	48812	+	GT/AG	2040
48251	48812	+	GT/AG	561
81034	81192	−	CT/AC	158
81034	85774	−	CT/AC	4740
81034	88414	−	CT/AC	7380
88553	88816	−	CT/AC	263
91080	91413	+	GT/AG	333
91123	91413	+	GT/AG	290
91247	91390	−	CT/AC	143
91318	91390	−	CT/AC	72
91553	92535	+	GT/AG	982
91553	97949	+	GT/AG	6396
91654	92433	−	CT/AC	779
97724	97949	+	GT/AG	225
97843	97949	+	GT/AG	106
1E + 05	1E + 05	+	GT/AG	1949
1E + 05	1E + 05	+	GT/AG	2426
1E + 05	1E + 05	+	GT/AG	358
1E + 05	1E + 05	−	CT/AC	138
1E + 05	1E + 05	−	CT/AC	135
1E + 05	1E + 05	−	CT/AC	764
1E + 05	1E + 05	+	GT/AG	167
1E + 05	1E + 05	+	GT/AG	609
1E + 05	1E + 05	+	GT/AG	202
1E + 05	1E + 05	−	CT/AC	211
1E + 05	1E + 05	−	CT/AC	230
1E+05	1E + 05	−	CT/AC	826

**Table 2:** Introns detected in five independent experiments.

33486	33634	+	GT/AG	148	x	x		x	x	x
41710	42239	+	GT/AG	529	x		x	x	x	x
47542	48031	+	GT/AG	489	x		x	x	x	x
53824	53869	+	GT/AG	45	x		x	x	x	x
69670	69923	+	GT/AG	253	x	x	x		x	x
79884	80090	+	GT/AG	206	x	x		x	x	x
81034	81383	−	CT/AC	349	x		x	x	x	x
81034	81642	−	CT/AC	608	x		x	x	x	x
81034	83364	−	CT/AC	2330	x	x	x		x	x
87740	88414	−	CT/AC	674	x		x	x	x	x
88553	90069	−	CT/AC	1516	x		x	x	x	x
89111	90069	−	CT/AC	958	x		x	x	x	x
91553	93625	+	GT/AG	2072	x	x	x		x	x
91553	94382	+	GT/AG	2829	x	x	x		x	x
123289	123507	−	CT/AC	218		x	x	x	x	x
123289	124570	−	CT/AC	1281		x	x	x	x	x
124151	124570	−	CT/AC	419		x	x	x	x	x
131183	132009	+	GT/AG	826		x	x	x	x	x
131183	132128	+	GT/AG	945	x		x	x	x	x
132354	132540	+	GT/AG	186	x		x	x	x	x
132373	132543	+	GT/AG	170	x	x		x	x	x
132640	133321	−	CT/AC	681	x		x	x	x	x
133903	135434	+	GT/AG	1531		x	x	x	x	x
134699	135211	−	CT/AC	512	x		x	x	x	x
134699	135814	−	CT/AC	1115	x		x	x	x	x
134699	136483	−	CT/AC	1784	x		x	x	x	x
134699	136600	−	CT/AC	1901	x		x	x	x	x
134699	137651	−	CT/AC	2952	x		x	x	x	x
135232	135339	+	GT/AG	107	x		x	x	x	x
137810	138985	−	CT/AC	1175	x		x	x	x	x
139059	139171	+	GT/AG	112	x		x	x	x	x
139059	139197	+	GT/AG	138	x		x	x	x	x
141330	141476	+	GT/AG	146	x		x	x	x	x
141330	142097	+	GT/AG	767	x		x	x	x	x
145646	145860	−	CT/AC	214	x	x		x	x	x
146105	147050	−	CT/AC	945	x		x	x	x	x

**Fig. 6:**
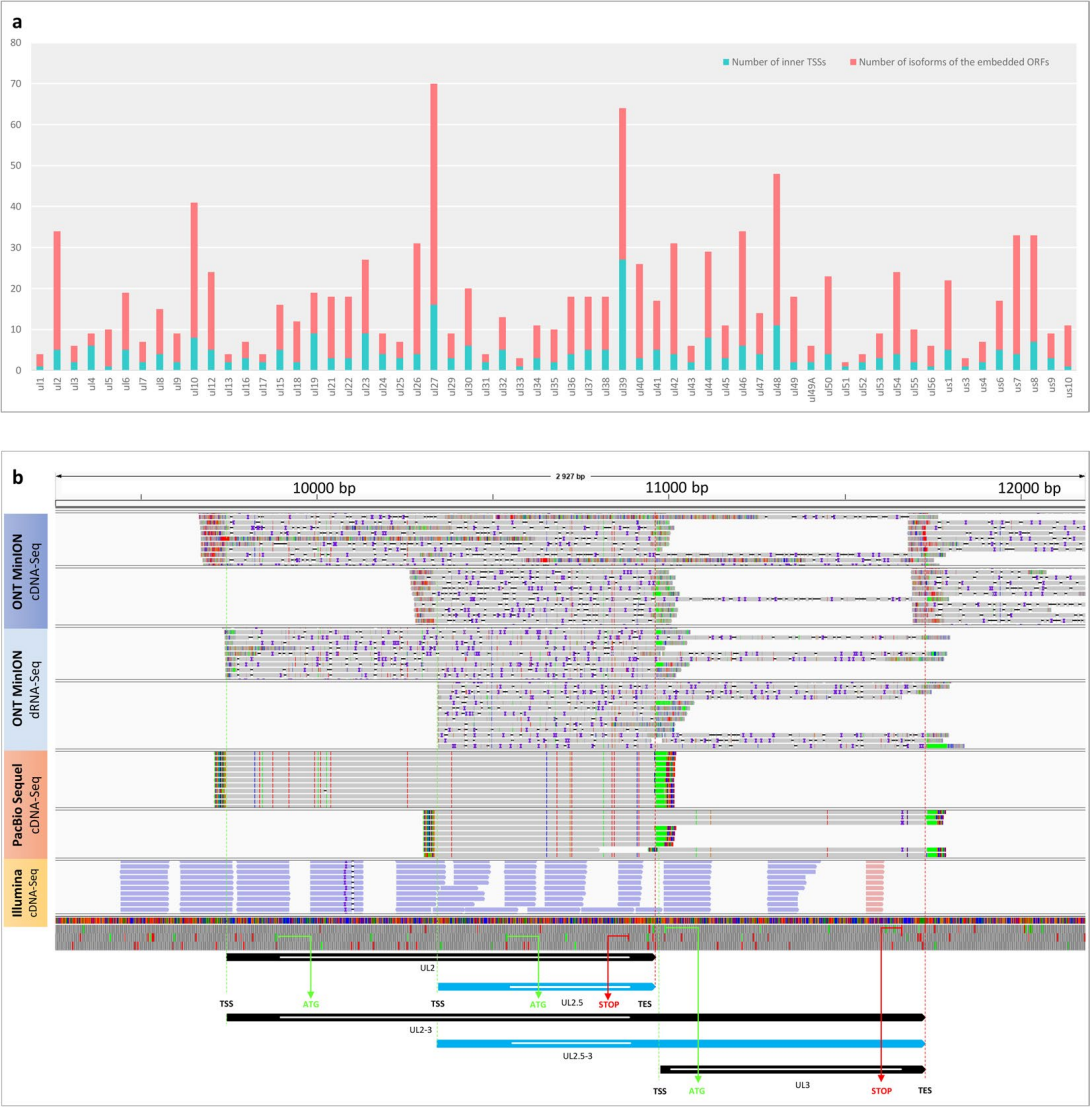
We have earlier published 63 embedded HSV genes (Tombácz *et al*. 2017). Sixty-one of them were validated using the dataset from Depledge’s publication. (**a)** Bar chart representation of the embedded ORFs. Many of the embedded ORFs have multiple length isoforms (Supplementary Table 2). (**b**) An example for an embedded ORF-containing transcript detected by various techniques. Visualization of the UL2 transcript and one of its truncated transcripts (ul2.5) using Integrative Genomics Viewer. The sequencing reads are from long-read (LRS) sequencing and short-read sequencing (SRS) datasets including direct RNA (dRNA) and cDNA sequencing. It can be seen that the dRNA-seq and the two LRS cDNA techniques detected the same TSS (note that dRNA sequencing produces shorter 5’-UTRs [on average, 23 bp are missing]). The figure also shows that SRS without a specialized library preparation method (e.g., CAGE) is not sufficient to identify 5’-ends of transcripts.

Additionally, we confirmed putative RNA molecules and transcript isoforms which were previously unpublished because of inadequate evidence supporting their existence (Supplementary Table 3). This meta-analysis also revealed that practically all HSV-1 genes contain at least one shorter transcript variant with 5′-truncated in-frame ORFs (Fig. 6). Loosening the annotation criteria probably would lead to the identification of truncated genes in every canonical gene. We also identified several fusion genes with relatively long introns spanning across gene boundaries (Supplementary Table 3b). We confirmed the RL2–UL1 and UL52–UL54 fusion transcripts described by Depledge and colleagues^19^ but the longer intron of the RL2–UL1 transcript was only present in a very low abundance in the remapped Illumina dataset published by Tang end co-workers^20^. However, it was undetected in the re-mapped, LoRTIA-filtered Depledge-dataset. In most fusion genes, only introns were identified but not precise transcript termini. Additionally, a large number of low-abundance transcript isoforms -including splice and length variants- were detected in this and other studies^28^ also. Whether these molecules have functional significance, or are merely the result of transcriptional noise remains to be ascertained. The general functions of embedded and fusion genes are also unknown. This work also revealed longer transcription start site (TSS) isoforms of several RNA molecules (Supplementary Table 3c). For example, we discovered longer TSS variants for the replication-associated RNAs (raRNAs)^29^ that overlap OriL or OriS (Fig. 2), and for latency-associated transcripts (LATs) (Fig. 2). The meta-analytic approach is also suitable for the elimination or addition of unconfirmed transcripts into the “putative” category. For example, a minor fraction of 5′- and 3′-truncated RNA molecules sequenced by the PacBio RS II platform^25^, were undetectable by other techniques, therefore they were removed from the latest list of HSV-1 transcripts.

Direct RNA sequencing is considered to be the golden standard of transcriptome analysis due its apparent lack of errors. However, we demonstrated here that this technique produces a relatively high level of errors. The fact that we could not detect a large number of dRNA introns in either cDNA database (205 introns in Depledge’s dataset and a single intron in our dRNA-Seq dataset) indicates lower than expected fidelity rate of dRNA-seq. In this study, the dRNA dataset produced the shortest average intron length. This dissimilarity in the two datasets is explained by the differences in the depths of coverage. However, the most abundant introns were present in databases of both approaches. Our meta-analysis confirmed the existence of an extremely complex meshwork of transcription overlaps (described by Tombacz and co-workers^27^), which is produced by transcriptional readthroughs between tandem and convergent genes and by the head-to-head overlap between divergent genes. Here, we identified several very long readthrough RNAs, including complex transcripts (containing at least two genes in opposite orientations), and transcript isoforms with long 5′-untranslated regions (5′-UTRs) (Supplementary Table 3c). Except for most parallel and some convergent overlaps, the majority of transcription readthroughs generate low-abundance transcripts the function of which, if any, is currently unknown. Transcriptional readthroughs might be the by-products of a genome-wide interference mechanism operating via the collision and competition of various elements of the transcription machinery^30^. This hypothesis does not exclude the possibility that the generated RNA strands also have functions of their own. Our comparative study clearly demonstrates the need for multiplatform and meta-analytic approaches for transcriptome profiling to obtain reliable results.

We assembled the sequence of HSV-1 transcripts using ReadConsensus script SeqTools (https://github.com/moldovannorbert/seqtools) and our previously published LRS data^27^. We found 157 transcripts after removing those with a read depth less than 30 × (Supplementary Table 4) Their sequence consensus can be found in *CITE*. We note here that *de novo* or reference-guided transcriptome annotation is more challenging than genome annotation due to the fact that the same DNA region generally codes for multiple RNA isoforms, including splice, TSS and TES variants. Higher read depths and multiple biological replicates are needed for such analyses.

## Discussion

In this study, we re-analysed and compared the datasets on HSV-1 transcriptome generated by eight studies^19–27^. Here, we provide the most complete transcriptome of HSV-1 to date. We identified a number of novel RNA molecules and transcript isoforms, including intron and length variants. We also confirmed the existence of previously published transcripts. This multiplatform study also identified and confirmed several low-abundance transcripts, such as mono- and multi-spliced transcripts, 5′-truncated mRNAs with short in-frame ORFs, and very long TSS variants, polycistronic and complex transcripts. The functions of these RNA molecules (if any) have to be demonstrated experimentally. Furthermore, we also demonstrated that various sequencing techniques, including dRNA-Seq, are error-prone, which can be circumvented by using integrated approaches. This study showed that using different reference genomes for mapping, the same transcripts can lead to somewhat different results with respect to the splice sites, especially in SRS. Taken together, employing multiplatform approaches with distinct library preparation methods is especially important in transcriptome research, because of the high error-rate and variance in the results obtained using various library preparation, sequencing and annotation methods. Furthermore, meta-analyses can account for the potential errors derived from using different kits and protocols, as well as from dissimilar work styles and conditions in different laboratories.

## Methods

*Datasets* In this study, several datasets (Depledge *et al*.^19^, Tombácz *et al*.^25,27^; Tang *et al*.^20^; Rutkowski *et al*.^21^, Whisnant *et al*.^22,23^ and Pheasant *et al*.^24^) were reanalysed to define the complete HSV-1 transcriptome. The datasets from our laboratory are filtered from data derived from PacBio and ONT cDNA sequencing, and various ONT library preparation approaches including cDNA-, Cap-selected cDNA and dRNA sequencing^26,27^. The wetlab and *in silico* protocols are detailed in the above mentioned studies. The SRS datasets were used only for the identification of intron donor and acceptor sites, whereas the LRS data were used to detect novel splice variants and TES- and TSS-isoforms.

**Fig. 7:**
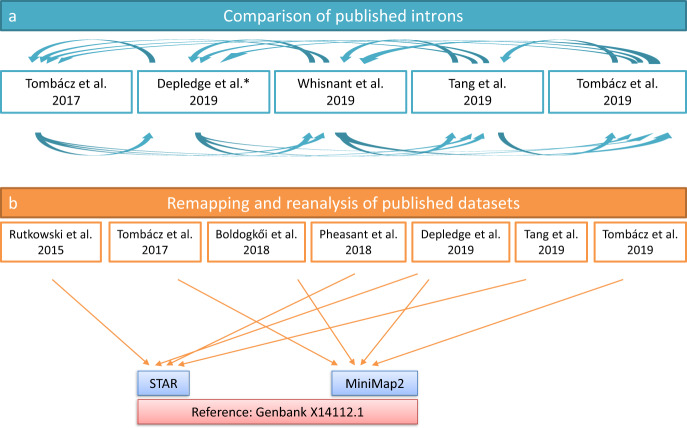
The network of datasets used for this meta-analysis study.

**Fig. 8:**
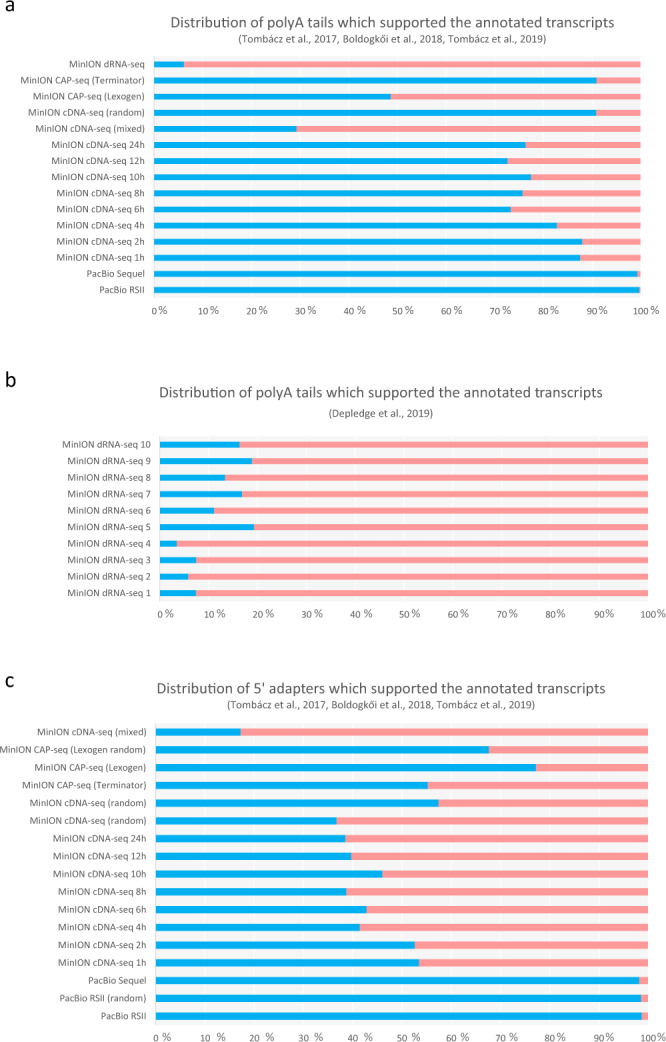
Distribution of LRS reads used for validation of TSS and TES positions. The horizontal bar graph shows the proportion of high quality/low quality adapter sequences of the LRS reads. **(a**) Proportion of the 3′-adapters within our dataset derived from various sequencing approaches utilised in our study^25,27^. The lowest ratios were obtained from MinION dRNA sequencing, and only a small amount (<10%) of the reads were used for the annotation/validation of TES positions; whereas the highest ratios were produced by the PacBio sequencing. (**b)** Proportion of the 3′-adapters in the dRNA-seq dataset from Depledge’s publication. Some of the parallel dRNA-seq experiments show a higher ratio compared to our dRNA-seq data. Still, the quality of adapters is substantially worse than the other approaches. (**c**) Proportion of the 5′-adapters within our dataset. The PacBio adapter reads 5′-end quality significantly better than any of the MinION methods.

*Data analysis* Detection of introns was carried out by a two-step analysis (Fig. 7). First, we compared published introns^19,20,22,26^ with each other, then we remapped the raw data and used them to identify potentially novel introns. We also analysed the effect of the selected reference genome and the aligner on the obtained results^19–27^ (Table 3). The adapter sequences from raw reads of each SRS run were removed using the Cutadapt v2.6 software. The fastp tool was used for validation. Next, we aligned the sequencing reads to the HSV-1 reference genome (GenBank: X14112.1) using minimap2 or STAR mapper for the LRS or the SRS data, respectively. The LoRTIA tool (https://github.com/zsolt-balazs/LoRTIA) was used to annotate introns, TSSs, and TESs from the LRS data (Fig. 8); whereas we used the STAR software to detect introns from the SRS samples. The previously published introns (Tang *et al*.^20^, Wishnant *et al*.^22^, and Tombácz *et al*.^25,27^) were compared with each other, reanalysed, and validated using the datasets from all of the aforementioned publications.

**Table 3:** Data records that were used in this study.

Data Record	Associated Paper	Database accession ID	Database
https://www.ebi.ac.uk/ena/data/view/PRJEB27861	Depledge *et al*.^19^	PRJEB27861	European Nucleotide Archive
https://www.ncbi.nlm.nih.gov/bioproject/PRJNA482043/	Tang *et al*.^20^	PRJNA482043	NCBI Sequence Read Archive
https://www.ncbi.nlm.nih.gov/bioproject/PRJNA483305	Tang *et al*.^20^	PRJNA483305	NCBI Sequence Read Archive
https://www.ncbi.nlm.nih.gov/bioproject/PRJNA533478	Tang *et* al.^20^	PRJNA533478	NCBI Sequence Read Archive
https://www.ncbi.nlm.nih.gov/geo/query/acc.cgi?acc=GSE59717	Rutkowsky *et al*.^21^ & Whisnant *et al*.^22,23^	GSE59717	Gene Expression Omnibus
https://www.ncbi.nlm.nih.gov/bioproject/PRJNA505045	Pheasant *et al*.^24^	PRJNA505045	NCBI Sequence Read Archive
https://www.ncbi.nlm.nih.gov/geo/query/acc.cgi?acc=GSE97785	Tombácz *et al*.^25^	GSE97785	Gene Expression Omnibus
https://www.ebi.ac.uk/ena/data/view/PRJEB25433	Boldogkői *et al*.^26^ Tombácz *et al*.^27^	PRJEB25433	European Nucleotide Archive

In this work, we assembled the sequence of HSV-1 transcripts with SeqTools/ReadConsensus scripts using our previously published LRS data^27^. The alignment of a transcript’s sequencing reads annotated by LoRTA to the section of the reference genome overlapped by the annotation was performed using minimap2. This was followed by variant calling using bcftools’ *mpileup* and *call* functions, and consensus sequence generation using bcftools’ *consensus* function. Read depth for each transcript was calculated by LoRTIA. To avoid sequencing errors, transcripts with a coverage of less than 30x were eliminated. This read depth is standard for MinION genome assembly^31–33^.

## Supplementary information

Supplementary Table 1

Supplementary Table 2

Supplementary Table 3

Supplementary Table 4

## Data Availability

The datasets used in this work were publicly available and were obtained from the original publications (Table 3): Depledge *et al*.^19^, Whisnant *et al*.^22,23^, Tang *et al*.^20^, Rutkowski *et al*.^21^, Pheasant *et al*.^24^, Boldogkői *et al*.^26^, and from Tombácz *et al*.^25,27^. All data generated in this study are included in Supplementary Tables 2 and 3. The data of introns plotted in this study were obtained from Tang *et al*.^20^, Rutkowski *et al*.^21^, and from Tombácz *et al*.^25,27^. The sequence of assembled transcripts was deposited under in Figshare^34^: 10.6084/m9.figshare.12057966.v2.

## Code availability

The codes for the LoRTIA and SeqTools (the toolkits developed by our laboratory) analysis are available at: https://github.com/zsolt-balazs/LoRTIA and https://github.com/moldovannorbert/seqtools, respectively.
